# Characteristics of exceptional responders to autologous stem cell transplantation in multiple myeloma

**DOI:** 10.1038/s41408-020-00353-8

**Published:** 2020-08-28

**Authors:** Ashley Paquin, Alissa Visram, Shaji K. Kumar, Morie A. Gertz, Hafsa Cantwell, Francis K. Buadi, Martha Q. Lacy, Angela Dispenzieri, David Dingli, Lisa Hwa, Amie Fonder, Miriam Hobbs, Suzanne R. Hayman, John A. Lust, Stephen J. Russell, Nelson Leung, Prashant Kapoor, Ronald S. Go, Yi Lin, Wilson I. Gonsalves, Taxiarchis Kourelis, Rahma Warsame, Robert A. Kyle, S. Vincent Rajkumar

**Affiliations:** 1 Mayo Clinic School of Medicine, Rochester, MN USA; 2 Mayo Clinic, Rochester, MN USA

**Keywords:** Epidemiology, Myeloma

## Abstract

Autologous stem cell transplantation (ASCT) is an important treatment modality in multiple myeloma (MM). However, relapse following ASCT is considered almost inevitable. This study aimed to characterize exceptional responders to ASCT, defined as progression-free survival (PFS) >8 years in the absence of maintenance therapy. We retrospectively analyzed patients treated at Mayo Clinic between August 1, 1998 and January 3, 2006, and included those with symptomatic MM, treated with an ASCT within 12 months of diagnosis. We found that 46 (9%) of the 509 patients who underwent ASCT during the study period were exceptional responders. The median duration of follow-up from diagnosis was 16.2 (interquartile range 14.3–17.7) years. The best response to therapy was a complete response (CR) or better in 34 (74%) of patients, and less than a CR in 12 (26%) of patients. The median PFS was 13.8 (95% confidence interval 10.5–18.5) years, and at the time of the last hematology assessment, 24 of 46 (52%) patients remained in remission. In conclusion, we showed that a small subset of patients with MM attains durable disease control without maintenance therapy post ASCT. Pre-emptive identification of these patients may help prevent undue toxicities and costs of subsequent therapy.

## Introduction

Multiple myeloma is a malignancy arising from neoplastic terminally differentiated plasma cell clones, and is the second most common hematologic malignancy in the United States^1^. Fortunately, the introduction of novel therapeutic agents, such as immunomodulatory drugs (IMIDs), proteasome inhibitors (PIs), and monoclonal antibodies, have significantly improved the overall survival (OS) of patients with multiple myeloma over the past two decades^2,3^. However, despite these advances, the majority of patients eventually relapse and experience shorter durations of disease-free remissions with each sequential line of therapy^4^. Despite the conventional belief that multiple myeloma remains an incurable malignancy, there have been a few reports of small proportions of patients maintaining prolonged periods of disease remission, raising the possibility that a subset of patients may be “functionally cured”^5–7^.

While disease prognosis is known to be affected by patient characteristics (i.e., age, functional status), baseline disease characteristics (i.e., renal function, cytogenetics, and gene expression profiles, β_2_-microglobulin, etc.), and response to therapy, specific characteristics that lead to a “functional cure” remain elusive^8–11^. In this study, our goal was to identify and characterize patients with an exceptional response to upfront autologous stem cell transplantation (ASCT). In a meta-analysis of randomized control trials comparing outcomes with or without lenalidomide maintenance post ASCT, there were no patients treated without maintenance therapy with ongoing disease remission beyond 100 months after ASCT, and the median progression-free survival (PFS) of patients treated without lenalidomide maintenance was 23.5 months^12^. Therefore, we defined exceptional responders as patients with disease-free survival of >96 months (8 years) post ASCT in the absence of maintenance therapy, which is ~4 times longer than what would be expected in this population.

## Methods

### Study cohort

We retrospectively assessed multiple myeloma patients treated at Mayo Clinic between August 1, 1998 and January 3, 2006. Patients were identified using the Mayo Clinic Multiple Myeloma database. Patients with symptomatic multiple myeloma (hypercalcemia, renal dysfunction, anemia, or lytic bone lesions) meeting the International Myeloma Working Group (IMWG) criteria of that era were included in this study if they underwent ASCT within 12 months of diagnosis and were followed locally with no evidence of disease progression for at least 96 months^13^. As maintenance therapy was not the standard of care during this time period, patients treated with maintenance regimens (not including zolendronic acid) were excluded. This study was approved by the Mayo Clinic Institutional Review Board.

### Data abstraction

Demographic, baseline laboratory and pathology studies, treatment, and disease response data were abstracted manually from the electronic medical record. All fluorescent in situ hybridization (FISH) studies were performed for clinical purposes at the Mayo Clinic, Rochester, as previously described^14,15^. Deletion of 17p was only considered if it was noted at initial diagnosis; all other cytogenetic abnormalities were considered if present at any time.

### Statistical analysis

Descriptive statistics were used to quantitate baseline characteristics. The Kaplan–Meier method was used to perform time to event analyses, including PFS, time to progression (TTP), and OS. PFS was defined as the date of diagnosis to either death or disease progression (as defined by the IMWG)^16^. TTP was defined as the time from diagnosis date to disease progression, and deaths were censored. Similarly, OS was defined as the date of diagnosis to death or last follow-up. The second PFS (PFS2) was defined as the time between the start of second-line therapy and either second disease progression or death. Univariable Cox proportional models were used to assess the hazard ratios and 95% confidence intervals (CIs) for risk factors associated with progression and OS. A two-sided *p* value of < 0.05 was considered to be significant. All statistical analyses were performed using JMP Pro v14.1 (SAS Institute, Cary, NC).

## Results

Five hundred and nine patients underwent an ASCT during the study period. We identified 51 patients with an exceptional response to therapy, defined as the absence of disease progression for at least 96 months. We excluded two patients because they received maintenance therapy post ASCT, and excluded three patients with progressive smoldering multiple myeloma that were treated prior to developing clear end-organ damage. Therefore, we identified 46 (9%) symptomatic multiple myeloma patients with an exceptional response to ASCT in the absence of maintenance therapy during the study period. The median duration of follow-up from diagnosis was 16.2 (interquartile range (IQR) 14.3–17.7) years.

The median age of patients at diagnosis was 57 (IQR 50–63) years. The majority of patients had low-risk disease, with 54% of patients classified as being an International Staging System (ISS) 1 at diagnosis. Two patients presented with extramedullary plasmacytomas at diagnosis. Cytogenetic data were available during the disease course for 40 of the 46 patients; however, FISH data were available for only 12 patients. One patient (2.5%) had high-risk cytogenetics with detectable t(14;16), seven (17.5%) had trisomies, and five (12.5%) had other isolated abnormalities. Only 37% of patients were treated with novel agents during induction; 16 (35%) patients were treated with a doublet including an IMID, and 1 (2%) patient was treated with a triplet including a PI and an IMID. Details on the conditioning regimen were available for 44 patients, all of whom received melphalan prior to ASCT. The median time from diagnosis to ASCT was 6 (IQR 5–8) months. Baseline characteristics of the 46 exceptional responder patients are further summarized in Table 1.

**Table 1:** Baseline characteristics of exceptional responders post ASCT.

	Patients (*n* = 46)
Median age at diagnosis, years (IQR)	57 (50–63)
Male, *n* (%)	18 (39)
Female, *n* (%)	28 (61)
Myeloma defining event	
Hypercalcemia, *n* (%)	6/43 (14)
Renal failure, *n* (%)	4/40 (10)
Anemia, *n* (%)	18/41 (44)
Osteolytic bone disease^a^, *n* (%)	27/43 (63)
>1 plasmacytoma, *n* (%)	4/46 (9)
Monoclonal protein isotype	
IgG, *n* (%)	30 (65)
IgA, *n* (%)	7 (15)
IgD, *n* (%)	1 (2)
Light chain, *n* (%)	8 (17)
Median bone marrow plasma cell percentage, *n* (IQR)	38 (19–68)
ISS (*n* = 35)	
1	19 (54)
2	11 (32)
3	5 (14)
Cytogenetics	
t(14;16), *n* (%)	1 (2.5)
Trisomy, *n* (%)	7 (17.5)
t(11;14), *n* (%)	2 (5)
Other isolated abnormalities, *n* (%)	5 (12.5)
No abnormality detected, *n* (%)	25 (62.5)
Induction therapy	
Thalidomide and dexamethasone, *n* (%)	16 (35)
Vincristine, doxorubicin, dexamethasone, *n* (%)	16 (33)
High-dose dexamethasone, *n* (%)	10 (22)
Melphalan and prednisone, *n* (%)	3 (6)
Bortezomib, thalidomide, dexamethasone, *n* (%)	1 (2)
Unknown, *n* (%)	1 (2)
ASCT conditioning	
Melphalan 200 mg/m^2^, *n* (%)	33 (72)
Melphalan 140 mg/m^2^, *n* (%)	4 (9)
Melphalan 200 mg/m^2^ + samarium, *n* (%)	4 (9)
Melphalan 200 mg/m^2^ + total body irradiation, *n* (%)	2 (4)
Melphalan 200 mg/m^2^ + ibritumomab tiuxetan, *n* (%)	1 (2)
Unknown	2 (4)

^a^Osteolytic bone disease was determined with the use of X-ray skeletal surveys at baseline.

Of 46 patients with response data available, the best response status was a complete response (CR) or better in 34 patients (74%), very good partial response (VGPR) in 6 patients (13%), partial response (PR) in 5 patients (11%), and stable disease in 1 patient (2%), as shown in Table 2. The median time to best response was 3 (IQR 0–4.5) months post ASCT. At the time of the last hematology assessment, 24 of 46 (52%) patients remained in remission; 19 patients had a continued CR, 1 person had a VGPR, 3 had PR, and 1 had stable disease. Peripheral blood testing for a minimal residual disease was only available for two patients who remained in remission at last follow-up; one patient was in a CR and was MRD negative (to a level of 10^–5^), and one patient with a stable disease was MRD positive. The one patient with t(14;16) at diagnosis was in a CR without prior disease progression at last follow-up, at 15.8 years post ASCT.

**Table 2:** Disease response during follow-up.

	Pre-transplant (*n* = 44)	Day + 100 post ASCT (*n* = 46)	Best response (*n* = 46)
CR or better	9 (20%)	27 (59%)	34 (74%)
VGPR	14 (32%)	13 (28%)	6 (13%)
PR	15 (34%)	5 (11%)	5 (11%)
Stable disease	6 (14%)	1 (2%)	1 (2%)

**Fig. 1:**
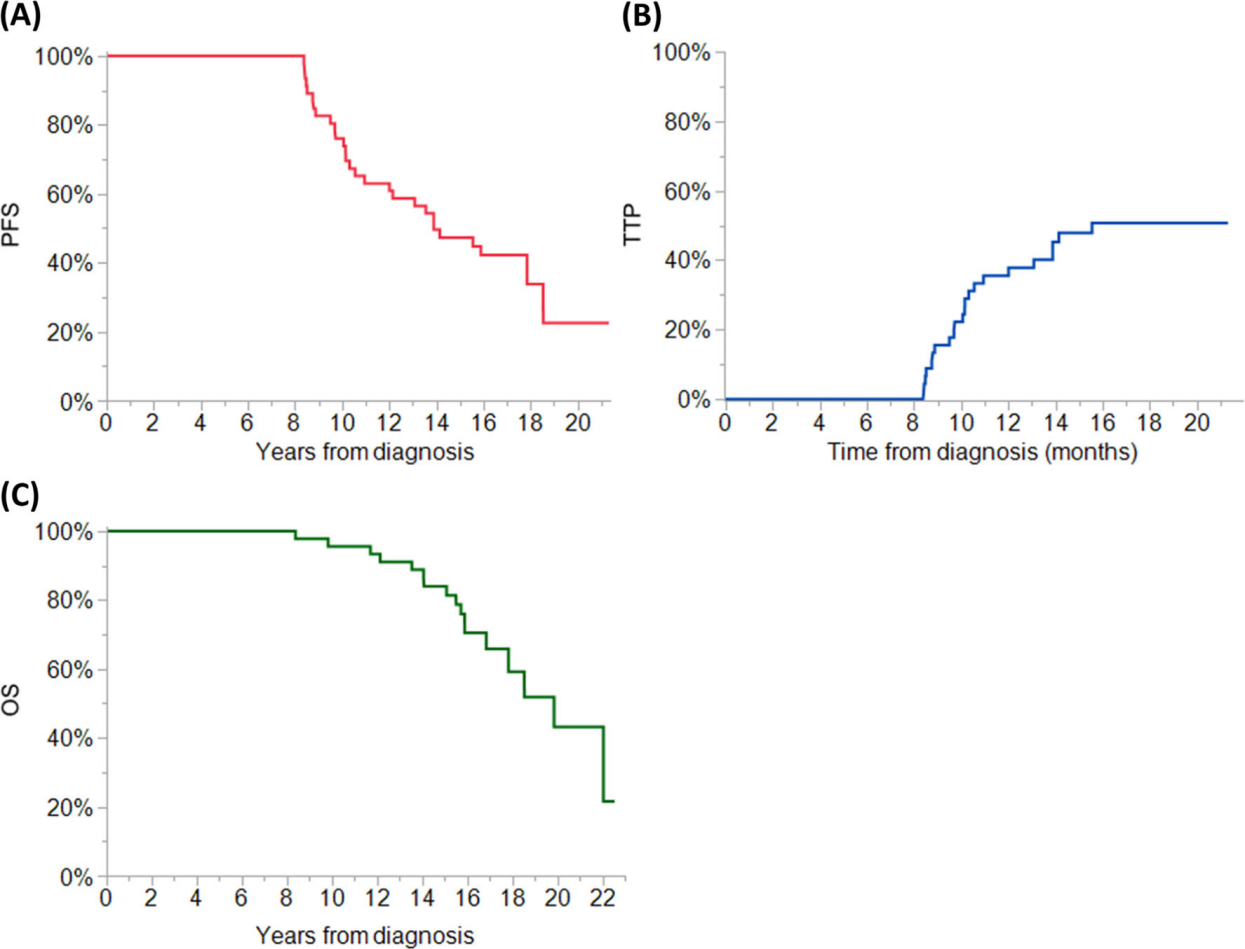
Outomes of Exceptional Responders to Autologous Stem Cell Transplantation (ASCT). For patients with an exceptional response post ASCT, PFS is shown in **a**, the TTP is shown in **b**, and OS is shown in **c**.

The median PFS was 13.8 (95% CI 10.5–18.5) years for the entire cohort (see Fig. 1a). In those patients achieving CR as the best response to ASCT, the median PFS was 15.5 (95% CI 12–NR) years. In the 22 patients who progressed during follow-up, the median PFS was 9.5 (95% CI 8.3–10.3) years, and the median PFS2 was 3.1 (95% CI 1.8–4.0) years. Disease progression was defined using the IWMG criteria (i.e., the reappearance of a detectable monoclonal protein if patients were in a CR), and only 5 of the 22 (23%) patients meeting the criteria for disease progression had end-organ dysfunction at that time^17^. Of the 22 patients with disease progression, 10 (45%) were observed until clinical relapse (the development of end-organ dysfunction attributable to disease), and this occurred at a median of 1.1 (0.5–4.4) years post hematologic progression. At disease relapse, 7 patients were treated with a second ASCT, 12 patients received IMIDs, and 6 patients received PIs. The median time to disease progression (TTP) for patients who progressed during follow-up was 10.1 (95% CI 8.8–10.9) years, and the median TTP overall was 15.6 (95% CI 10.9–NR) years (see Fig. 1b). The TTP curve did appear to plateau after 16 years of follow-up, whereas there was no plateau observed in the PFS curve. In the PFS analysis, three patients met the criteria for PFS due to death after 15 years of follow-up, and these patients would have been censored in the TTP analysis. However, in two of the three patients who died after 15 years of follow-up, the cause of death was not documented and clinic records were not available in the year preceding death, so death due to myeloma recurrence cannot be ruled out.

The median OS post ASCT was 19.9 (95% CI 16.8–NR) years for the entire cohort (see Fig. 1c) During the course of follow-up, 17 (37%) patients died; 3 from refractory multiple myeloma (2 of these patients acquired del(17p) at the time of relapse), 2 from metastatic secondary malignancy (ovarian adenocarcinoma and melanoma, respectively), 2 from therapy-related acute myeloid leukemia, 1 from complications of engraftment failure following salvage ASCT for relapsed multiple myeloma, 1 from complications of amyloid cardiomyopathy, and 1 from septic shock. Details on the cause of death were not found for seven patients.

**Fig. 2:**
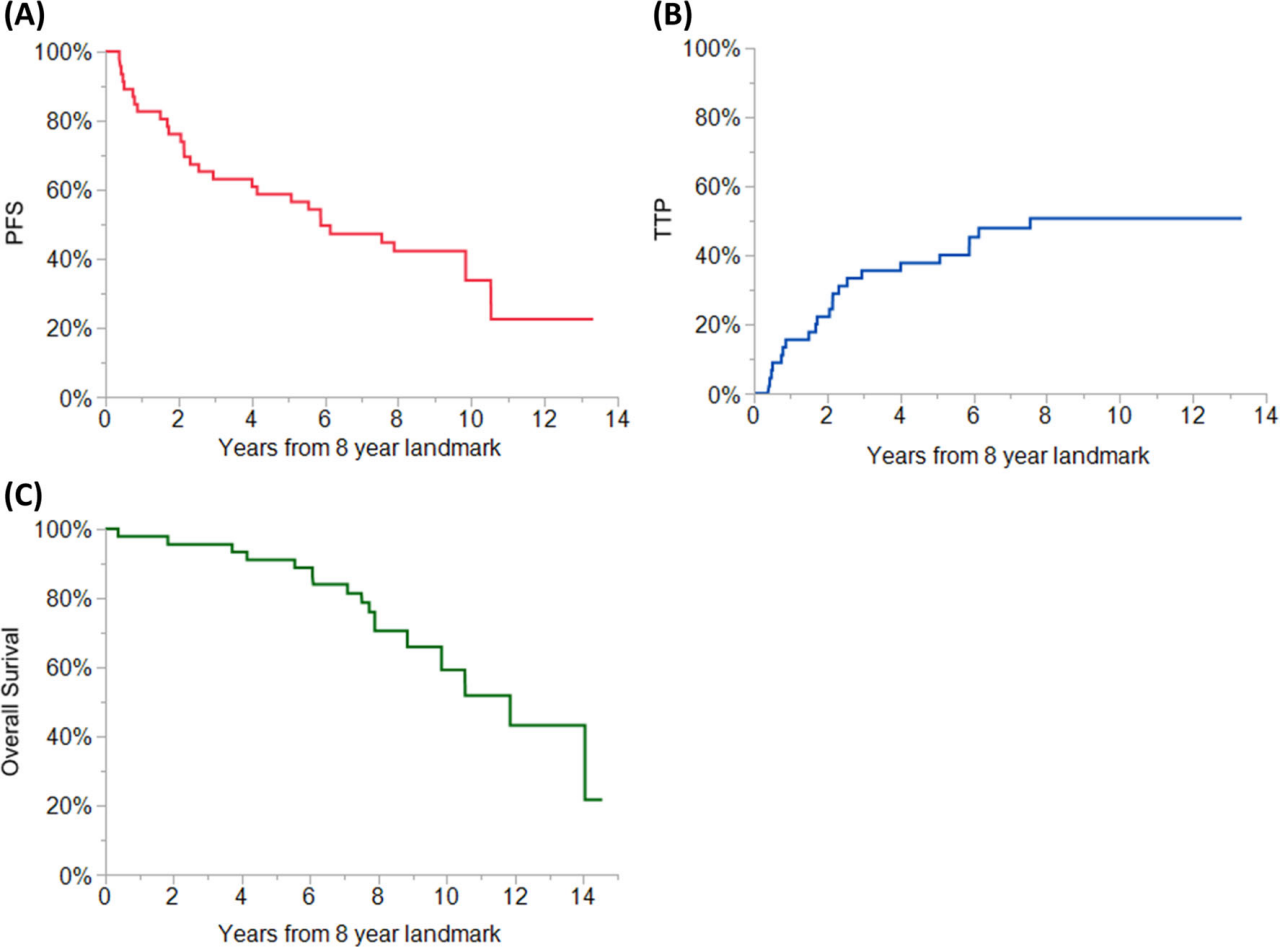
Landmark Analysis showing Outcomes of Exceptional Responders to Autologous Stem Cell Transplantation (ASCT). A landmark analysis at 8 years after diagnosis of multiple myeloma showing the PFS (**a**), TTP (**b**), and OS (**c**) of exceptional responder patients.

From the landmark time of 8 years, the median TTP was 7.6 (95% CI 2.9–NR) years, and the median OS was 11.9 (95% CI 8.8–NR) years (as shown in Fig. 2).

A univariate Cox proportional hazards analysis was performed in order to identify factors associated with improved PFS and OS among exceptional responder patients. Although the age ≥65 years significantly increased the risk of disease progression or death (hazard ratio (HR) 3.5, 95% CI 1.36–9.05, *p* = 0.02), it was not associated with a significant improvement in OS. Similarly, baseline patient and disease characteristics (gender, ISS at diagnosis, plasma cell percentage in the bone marrow at diagnosis, monoclonal protein isotype, lactate dehydrogenase (LDH) at diagnosis, and renal failure at presentation), type of induction therapy, and disease response did not significantly affect PFS or OS (see Table 3). There was no difference in OS between patients who progressed vs. those who were in remission at the time of last follow-up (HR 2.13, 95% CI 0.77–5.93, *p* = 0.139).

**Table 3:** Univariable Cox proportional hazard analysis of variables that affect progression-free survival and overall survival in patients with an exceptional response to therapy post ASCT.

	PFS		OS	
	HR (95% CI)	*P* value	HR (95% CI)	*P* value
Age ≥65 vs. age <65	3.53 (1.36–9.05)	**0.02**	1.97 (0.54–7.13)	0.336
ISS 1 vs. 2/3	0.65 (0.28–1.55)	0.334	1.06 (0.37–3.08)	0.904
Male vs. female	1.49 (0.71–3.17)	0.296	2.47 (0.94–6.53)	0.065
Plasma cell percentage at diagnosis ≥40% vs. <40%	1.47 (0.66–3.27)	0.329	0.99 (0.36–2.75)	0.987
IgA vs. non-IgA monoclonal protein	0.91 (0.31–2.63)	0.859	0.24 (0.03–1.84)	0.091
LDH >222 vs. normal	0.82 (0.23–2.92)	0.758	0.85 (0.13–3.32)	0.837
Renal failure at diagnosis vs. no renal failure	0.92 (0.15–3.20)	0.913	4.74 (0–NR)	0.114
IMID vs. non-IMID induction	1.16 (0.52–2.61)	0.719	1.47 (0.48–4.53)	0.508
At day +100 post ASCT, CR vs. less than CR	1.09 (0.50–2.37)	0.82	1.83 (0.59–5.66)	0.268
Best response CR vs. non-CR	0.47 (0.21–1.05)	0.081	0.90 (0.29–2.81)	0.859
Disease progression vs. no disease progression	–		2.13 (0.77–5.93)	0.139

*NR* not reached.

## Discussion

In this study, we describe that 9% of multiple myeloma patients treated with upfront ASCT had a disease remission of >8 years even without maintenance therapy. The median PFS was 13.8 years, which is almost seven times longer than expected in this population^12^.

The median age of exceptional responder patients in this study was 57 years. While this is younger than the average transplant-eligible patient in the current era, the treatment paradigm during the study period was that ASCT was limited to patients <65 years of age^17,18^. The number of patients with renal failure at diagnosis was also lower than would be expected when compared to historical data published from our institution^8^. However, this may be a reflection of the fact that patients eligible for transplant are younger, and generally have fewer comorbidities. There was also only one patient (2.5% of the exceptional responder population) with an identified high-risk cytogenetic abnormality, t(14;16), which is much lower than expected^10^. The lack of high-risk cytogenetics was also reported in two studies of patients with prolonged disease-free remission after frontline therapy^6,19^. However, it is important to note that a minority of patients (*n* = 12, 26%) in this cohort had FISH performed, as this was not the standard of care at the time. Therefore, the lack of patients with high-risk cytogenetics may reflect the decreased sensitivity of conventional cytogenetics at detecting chromosomal aberrations. Otherwise, baseline demographics, disease characteristics, and clinical presentation were similar to what has been reported in the general population with multiple myeloma^8^.

The notion that a small proportion of patients with multiple myeloma can have long disease-free remissions, potentially a “functional cure”, has been demonstrated in other studies. Long-term follow-up of the total therapy programs, which maximize upfront therapies with the aim of eradicating the drug-resistant cancer cell clones through multiple mechanisms of action, show that with intensive therapy the TTP curves plateau after ~10 years in patients with a low-risk gene expression profile^5,20^. However, prolonged PFS rates have been reported even without the use of intensive therapies; Vu et al.^19^ showed that 14% (*n* = 33) of patients treated with first-line lenalidomide maintained a PFS >6 years, even though only 27% of those patients received an upfront ASCT. Similarly, Terpos et al.^6^ reported that 9% (*n* = 36) of newly diagnosed multiple myeloma patients experienced a PFS >7 years, with the majority (70%) of long-term responders receiving IMID- or PI-based therapy, and 60% of patients receiving an upfront ASCT.

In this study, we found that there appears to be a plateau in the TTP curve 16 years after diagnosis without any ongoing therapy post ASCT. While this may seem to support the theory that a functional cure is obtainable, only 16 (35%) exceptional responder patients were followed beyond 16 years, for a median additional follow-up of 1.2 years. Importantly, this study shows that even after long periods of disease remission, disease progression does occur. In those that progressed, the median PFS2 was much shorter than the PFS from diagnosis (9.5 vs. 3.1 years, respectively), indicating that the underlying disease biology in exceptional responder patients is not indolent indefinitely. Therefore, further follow-up is required to assess the durability of remission in the exceptional responders who have not progressed to date, to assess whether these patients are truly “cured.”

Prior studies have described patient and disease characteristics that are associated with an exceptional disease-free remission in patients with multiple myeloma^6,7,19–21^. One of the consistent findings has been that achieving a deeper response, such as a CR or MRD-negative status, is associated with improved outcomes^7,21–23^. The association of MRD negativity with improved depth of disease response and PFS has been the rationale for intensifying the treatment of newly diagnosed multiple myeloma patients by using quadruplet regimens^24–26^. In this study we attempted to identify factors that would stratify exceptional responders based on their PFS or OS; although we did not find a significant difference in any baseline patient or disease characteristics that predicted superior PFS or OS outcomes, including depth of response to therapy, this analysis was likely limited by the small number of exceptional responders in this study. Interestingly, similar to the findings of Vu et al.^19^, in this study, ~25% of exceptional responders achieved that status despite not being in CR at any time. The median PFS in these patients was 10.2 years without antimyeloma therapy post ASCT. Although this may seem paradoxical, exceptional responders have been shown to have alterations in the bone marrow microenvironment that may allow for more robust antitumor immune surveillance^27,28^. Furthermore, the gene signature of myeloma patients surviving >10 years after initiating therapy has been shown to be similar to patients with monoclonal gammopathy of undetermined significance (MGUS). Myeloma patients with an “MGUS-like” gene signature were less likely to attain a complete remission when compared to myeloma patients without an MGUS-like signature, but more likely to have an improved 5-year survival^29^. Our study shows that for a subset of myeloma patients, CR- or MRD-negative status may not be required in order to achieve a prolonged duration of remission even without other therapy.

In conclusion, we showed that 9% of patients with newly diagnosed multiple myeloma treated with an upfront ASCT have a PFS of >8 years without maintenance or other subsequent therapy. Approximately 25% of these patients achieved durable remissions even without obtaining a CR, reinforcing the need to move away from a “one size fits all” approach to treating myeloma. Our study shows that a subset of patients with an exceptional response may be “functionally cured” based on a plateau in TTP curves on long-term follow-up. Further studies are needed to pre-emptively identify this subset of patients who may not need aggressive, life-long therapy to maintain disease control, in order to decrease the long-term risk of financial and physical toxicity. Finally, although the goal of this study was to determine the proportion of patients who achieve exceptional results with stem cell transplantation alone, we believe that post-transplant maintenance improves OS and this study should not be seen as questioning the need for such treatment.
